# Neurological Effects of Honey: Current and Future Prospects

**DOI:** 10.1155/2014/958721

**Published:** 2014-04-27

**Authors:** Mohammad Mijanur Rahman, Siew Hua Gan, Md. Ibrahim Khalil

**Affiliations:** ^1^Department of Biochemistry and Molecular Biology, Jahangirnagar University, Savar, Dhaka 1342, Bangladesh; ^2^Human Genome Centre, School of Medical Sciences, Universiti Sains Malaysia, 16150 Kubang Kerian, Kelantan, Malaysia

## Abstract

Honey is the only insect-derived natural product with therapeutic, traditional, spiritual, nutritional, cosmetic, and industrial value. In addition to having excellent nutritional value, honey is a good source of physiologically active natural compounds, such as polyphenols. Unfortunately, there are very few current research projects investigating the nootropic and neuropharmacological effects of honey, and these are still in their early stages. Raw honey possesses nootropic effects, such as memory-enhancing effects, as well as neuropharmacological activities, such as anxiolytic, antinociceptive, anticonvulsant, and antidepressant activities. Research suggests that the polyphenol constituents of honey can quench biological reactive oxygen species and counter oxidative stress while restoring the cellular antioxidant defense system. Honey polyphenols are also directly involved in apoptotic activities while attenuating microglia-induced neuroinflammation. Honey polyphenols are useful in improving memory deficits and can act at the molecular level. Therefore, the ultimate biochemical impact of honey on specific neurodegenerative diseases, apoptosis, necrosis, neuroinflammation, synaptic plasticity, and behavior-modulating neural circuitry should be evaluated with appropriate mechanistic approaches using biochemical and molecular tools.

## 1. Introduction

Honey, a natural food product, is a sweet, viscous substance that is formed from the nectar of flowers by honeybees (*Apis mellifera*; Family: Apidae). The conversion of nectar to honey is an impressively complex process. Nectar is first collected from flowers and undergoes ripening by partial enzymatic digestion in the honey stomach of the honeybee. The ripened nectar is then matured by moisture evaporation through fanning by the bees, which leaves a moisture content of only approximately 13 to 18% in the honey [1]. Honey has been utilized by humans since prehistoric times, before civilization appeared approximately 5,500 years ago. Most ancient civilizations, such as the Egyptians, Greeks, Chinese, Mayans, Romans, and Babylonians, used honey both for nutritional purposes and for its medicinal properties [2]. Honey is the only insect-derived natural product, and it has therapeutic, religious, nutritional, cosmetic, industrial, and traditional value.

The global production of honey increased by 10%, from 1,419,072 to 1,555,980 tons, between 2005 and 2010 [3]. In addition to the consumption of raw honey, the use of honey in beverages is also increasing in popularity. Although modern science has reported its medical benefits, honey has historically been utilized in various food products as a sweetening agent and in medicine as a therapeutic agent for wound healing and for the treatment of cataracts [2, 4]. Raw honey has been used for centuries by traditional medical practitioners worldwide in numerous medical treatments, such as treatments for eye diseases in India, cough and sore throat in Bangladesh, leg ulcers in Ghana, and measles in Nigeria [5].

The traditional knowledge of honey and modern science are merged in “apitherapy,” which denotes the medical use of honey and bee products. Apitherapy has become a major focus of research involving alternative medicine because a wide variety of well-known preventive or curative methods from folk medicine use honey to treat different ailments, and the therapeutic properties of honey have been increasingly documented in the modern scientific literature [6–8]. Recently, the oral ingestion of raw honey has been indicated for insomnia, anorexia, stomach and intestinal ulcers, constipation, osteoporosis, and laryngitis. Externally applied honey is used to treat athlete's foot, eczema, lip sores, and both sterile and infected wounds caused by accidents, surgery, bedsores, or burns. In many countries, including France and Germany, physicians recommend using honey as a first-line treatment for burns, superficial wounds, and in some cases, even deep lesions such as abscesses [9].

## 2. Nutritional Facts about Honey

To date, approximately 300 varieties of honey have been identified [5]. These varieties exist due to the variable types of nectar that are collected by the honeybees. Although there have been many nutritional studies of honey, only a few are representative. Carbohydrates are the main constituents of honey and contribute 95 to 97% of its dry weight. In addition to carbohydrates, honey contains numerous compounds, such as organic acids, proteins, amino acids, minerals, and vitamins [10, 11] (Table 1). Pure honeys were also reported to contain polyphenols, alkaloids, anthraquinone glycosides, cardiac glycosides, flavonoids, reducing compounds, and volatile compounds [12–14].

Monosaccharides, such as fructose and glucose, are the predominant sugars present in honey, and they are said to be responsible for most of the physical and nutritional characteristics of honey [15]. Smaller quantities of other types of sugars, such as disaccharides, trisaccharides, and oligosaccharides, are also present in honey. The disaccharides primarily include sucrose, galactose, alpha,beta-trehalose, gentiobiose, and laminaribiose, whereas the trisaccharides primarily include melezitose, maltotriose, 1-ketose, panose, isomaltose glucose, erlose, isomaltotriose, theanderose, centose, isopanose, and maltopentaose [15–17]. Approximately 5 to 10% of total carbohydrates are oligosaccharides, and approximately 25 different oligosaccharides have been identified [18, 19]. Many of these sugars are not found in the nectar but are formed during the honey ripening and maturation phases.

Gluconic acid, which is a product of glucose oxidation by glucose oxidase, is the major organic acid that is found in honey; in addition, minor amounts of formic, acetic, citric, lactic, maleic, malic, oxalic, pyroglutamic, and succinic acids have also been detected [20]. These organic acids contribute to the acidic (pH between 3.2 and 4.5) characteristic of honey [21]. However, honey can also behave as a buffer.

Honey also contains several physiologically important amino acids, including all nine essential amino acids and all nonessential amino acids except for glutamine and asparagine. Among the amino acids present, proline is predominant, followed by aspartate, glutamate, and some other types of amino acids [22]. However, in another study, proline was reported as the primary amino acid in honey, followed by lysine [23]. Enzymes that are either secreted from the hypopharyngeal glands of the bee or originate from the botanical nectars constitute the main protein component of honey. These enzymes include the bee hypopharyngeal gland-derived diastase (an amylase that digests starch to maltose), invertases (e.g., saccharase and *α*-glucosidase that catalyzes the conversion of sucrose to glucose and fructose), glucose oxidase (which produces hydrogen peroxide and gluconic acid from glucose), and plant-derived catalase (which regulates the production of hydrogen peroxide), along with acid phosphatase [24].

The vitamin content in honey is generally low and does not meet the recommended daily intake (RDI). Usually, all of the water-soluble vitamins are present in honey, with vitamin C being the most abundant. Approximately 31 different minerals have been detected in honey, including all of the major minerals, such as calcium, phosphorus, potassium, sulfur, sodium, chlorine, and magnesium (Table 2). Some essential trace minerals are also reported to be present in honey, such as rubidium (RB), silicon (Si), zirconium (Zr), vanadium (V), lithium (Li), and strontium (Sr), as well as some trace elements, such as lead (Pb), cadmium (Cd), and arsenic (As), which could be present due to contaminants from surrounding environments [25]. Interestingly, the amounts of these minerals follow a geographical variation; the mineral compositions of honeys that are collected from similar regions are similar. However, several previous reports claimed that honey is a poor source of minerals [8, 22], whereas several other recent reports suggest that honey is rich in minerals [26, 27]. Essential trace elements are important, particularly among growing children because of their rapid growth and development. Nevertheless, a comparison with the RDI clearly indicates that honey contains a substantial amount of several essential trace elements that would partially meet the RDI for children (Table 2). For adults, honey is a good source of potassium.

## 3. Other Nonnutritional Components of Honey

Previous studies have reported the presence of approximately 600 different volatile compounds in honey, and these compounds can be used to characterize its botanical source [28]. In addition, volatile compounds can also impart aromatic characteristics to honey and contribute to its potential biomedical activity [28]. The volatile composition of honey is generally low but includes hydrocarbons, aldehydes, alcohols, ketones, acid esters, benzene and its derivatives, furan and pyran, norisoprenoids, terpene and its derivatives, and sulfur, as well as cyclic compounds [29, 30].

Polyphenols and flavonoids, which act as antioxidants, are two important bioactive molecules that are present in honey. Emerging evidence from recent studies has confirmed the presence of approximately 30 different polyphenols in honey [31, 32]. The total polyphenol content of honey varies from 50 to 850 mg/kg, whereas the flavonoid content varies from 36 mg/kg to 150 mg/kg [12, 33, 34]. The presence and concentrations of these polyphenols in honeys can vary depending upon the floral source and the geographical and climatic conditions. Some bioactive compounds, such as Galangin, kaempferol, quercetin, isorhamnetin, and luteolin, are present in all types of honey, whereas others, such as hesperetin and naringenin, are reported only in specific varieties [35]. Overall, the most commonly reported phenolic and flavonoid compounds in honey include ellagic acid, gallic acid, syringic acid, benzoic acid, cinnamic acid, ferulic acids, chlorogenic acid, caffeic acid, coumaric acid, myricetin, chrysin, hesperetin, isorhamnetin, quercetin, galangin, apigenin, catechin, kaempferol, naringenin, and luteolin [7, 31, 32].

## 4. Effects of Honey on Brain Structures and Functions

### 4.1. Current Experimental Evidence of the Nootropic and Neuropharmacological Effects of Honey

Research from the past two decades has explored honey as an enigmatic gel that has gastroprotective, hepatoprotective, reproductive, hypoglycemic, antioxidant, antihypertensive, antibacterial, antifungal, anti-inflammatory, immunomodulatory, wound healing, cardio-protective and antitumor effects [6, 26, 36, 37]. Unfortunately, research on the nootropic and neuropharmacological effects of honey is scarce. Nevertheless, the belief that honey is a memory-boosting food supplement is actually ethnotraditional as well as ancient in nature. For instance, honey is reported to be an important component of Brahma rasayan, an Ayurvedic formulation that is prescribed to extend the lifespan and improve memory, intellect, concentration, and physical strength [38].

One established nootropic property about honey is that it assists the building and development of the entire central nervous system, particularly among newborn babies and preschool age children, which leads to the improvement of memory and growth, a reduction of anxiety, and the enhancement of intellectual performance later in life [26]. Additionally, the human brain is known to undergo postnatal development with the obvious maturation and reorganization of several structures, such as the hippocampus and cerebral cortex. It has been reported that this postnatal development occurs through neurogenesis, which occurs predominantly during childhood, and this development can also extend into adolescence and even through adulthood [39]. Empirical, but striking, evidence supporting this concept was provided by an experiment that was conducted on postmenopausal women; those who received honey showed improvements in their immediate memory but not in immediate memory after interference or in delayed recall [40]. In another study, the normal diet of two-month-old rats was supplemented with honey, and their brain function was assessed over a one-year period. Honey-fed rats showed significantly less anxiety and better spatial memory throughout all stages compared with the control group of rats. More importantly, the spatial memory of honey-fed rats, as assessed by object recognition tasks, was significantly greater during later months (i.e., 9 and 12) [41].

In agreement with the previous study, both short- and long-term supplementations with honey at a dose of 250 mg/kg body weight significantly decreased the lipid peroxidation in brain tissue with a concomitant augmentation of superoxide dismutase (SOD) and glutathione reductase activity. Thus, honey consumption ameliorates the defense mechanism against oxidative stress and attenuated free radical-mediated molecular destruction [39]. Furthermore, honey decreased the number of degenerated neuronal cells in the hippocampal CA1 region, a region that is known to be highly susceptible to oxidative insult [42]. Theoretically, the cumulative macromolecular destruction by free radicals due to an imbalance between the prooxidant and antioxidant defense systems is implicated in aging [43]. Many studies have focused on the evidence of oxidative stress in neurodegenerative diseases, such as Alzheimer's disease (AD), mild cognitive impairment, Parkinson's disease (PD), amyotrophic lateral sclerosis (ALS), and Huntington's disease (HD) [44].

Emerging research has documented the neuropharmacological effect of honey as a nutraceutical. Oyekunle et al. [45] conducted the first such study, in which rats were fed with different concentrations of honey (10, 20 and 40%) at a dose of 0.5 mL/100 g. Significant dose-dependent increases in exploratory activities in a hole board test and in locomotor, rearing and grooming activities in an open-field test were found in the honey-fed test groups rats compared with the control group rats. These findings indicate that the consumption of honey mitigates anxiety and exerts an excitatory effect on the central nervous system, especially at the highest nonsedative dose [45]. In another study, the neurological effects of honey were investigated by assessing spatial working memory in mice using (1) the Y-maze test and (2) pentobarbital-induced hypnosis and assessing, (3) its anxiolytic activities using hole-board and elevated plus-maze tests, (4) its anticonvulsant activity in an picrotoxin seizure model, (5) its antinociceptive activity in hot-plate and tail-flick tests, and (6) its antidepressant effects using the forced swimming test. The authors of that study concluded that honey is a functional food that possesses anxiolytic, antinociceptive, anticonvulsant, and antidepressant effects [46].

Indeed, the neuropharmacological impact of honey reflects the preliminary modulatory ability of the neural circuit and associated neurochemical systems that underlie the behavioral and molecular changes associated with the experimental paradigm. These insights into the neuropharmacological effects of honey highlight the neurological factors that are influenced by treatment with honey. Exploratory behaviors often involve the excitatory neural systems, such as the cholinergic and dopaminergic systems, whereas anxious behavior often involves the inhibitory neural system, specifically *γ*-aminobutyric acid (GABA) [47–49]. Several lines of experimental evidence support the hypothesis that the neuropharmacological effects of honey are mediated via dopaminergic and nonopioid central mechanisms, such as the voltage-gated sodium channel blocking hypothesis, the activation of the noradrenergic inhibitory system and/or serotonergic systems, and the GABAergic system [45, 50].

In addition to neural effects, glial cells may also respond to honey therapy because honey shows a neuroprotective effect in the cerebral focal-induced ischemia model in rats [51]. Moreover, honey attenuated ischemia-induced neuroinflammation by activating microglia, and neuroinflammatory processes in the brain are believed to play a crucial role in the development of neurodegenerative diseases as well as in neuronal injury associated with stroke [52, 53]. Interestingly, ischemia-induced cognitive impairments that result from microglia- and/or astrocyte-mediated neuroinflammation were also significantly attenuated by honey therapy [52, 54].

## 5. The Effects of Physiologically Active Moieties in Honey on Brain Function

Oxidative stress is a common manifestation of all types of biochemical insults to the structural and functional integrity of neural cells, such as aging, neuroinflammation, and neurotoxins. The brain is highly susceptible to oxidative damage due to its high oxygen demand as well as to the high amount of polyunsaturated fatty acids (PUFAs) in the neuronal membranes [55]. Different phytochemical compounds have been shown to have scavenging activities and can activate key antioxidant enzymes in the brain, thus breaking the vicious cycle of oxidative stress and tissue damage [56, 57]. Several supplementary research reports have suggested that the neuroprotective effect of the polyphenols present in honey involves several important activities within the brain. These effects include protection against oxidative challenge; the attenuation of neuroinflammation; the promotion of memory, learning, and cognitive function; and protection against neurotoxin-induced neuronal injury. We describe several important constituents in honey that may play this protective role.

Apigenin is a common flavonoid that is frequently identified in honey. In addition to its radical scavenging activity, apigenin protects neurons against oxygen-glucose deprivation/reperfusion-induced injury in cultured primary hippocampal neurons by improving sodium/potassium-ATPase (Na^+^/K^+^-ATPase) activities [58]. Apigenin also inhibits the kainic acid-induced excitotoxicity of hippocampal cells in a dose-dependent manner by quenching reactive oxygen species and by inhibiting the depletion of reduced glutathione (GSH) levels [59]. Apigenin suppresses the interferon gamma (IFN-*γ*)-induced expression of CD40, whereas the signaling of CD40 is critically involved in microglia-related immune responses in the brain. Rezai-Zadeh et al. suggested that apigenin may have neuroprotective and disease-modifying properties in several types of neurodegenerative disorders [60]. Moreover, apigenin stimulates the adult neurogenesis that underlies learning and memory [39].

Caffeic acid, another important antioxidant, is a type of phenolic acid that is present in honey, as well as in coffee, fruits and vegetables. An* in vitro* study has demonstrated the neuroprotective effects of caffeic acid on neuronal cells [61]. The neuroinflammatory suppression activity of caffeic acid can be inferred from the observation that caffeic acid reverses the aluminum-induced overexpression of 5-lipoxygenase (5-LOX) in brain tissues [62]. Caffeic acid also prevents the aluminum-induced damage of the cerebrum that is associated with neuronal death in the hippocampus and with learning and memory deficits [62].* In vitro* treatment with caffeic acid at several different concentrations has been reported to increase the acetylcholinesterase activity in the cerebral cortex, cerebellum, and hypothalamus. A similar scenario is also observed in the cerebellum, hippocampus, hypothalamus, and pons when caffeic acid is administered* in vivo*. All of these findings strongly support the proposition that caffeic acid improves memory by interfering with cholinergic signaling, in addition to its neuroprotective effects [63].

Catechin is a flavonoid that contributes to the antioxidant activities of honey. Several studies have repeatedly demonstrated the neuroprotective effects of catechin on neuronal death in a wide array of cellular and animal models of neurological diseases [64, 65]. Although catechin possesses potent iron-chelating, radical-scavenging, and anti-inflammatory activities, current studies have indicated that the modulation of signal transduction pathways, cell survival, or death genes and mitochondrial function significantly contribute to the induction of cell viability [66]. For instance, according to Unno et al., the daily consumption of green tea, which contains high levels of catechin, can delay the memory regression that is associated with age-related brain atrophy and cognitive dysfunction [67]. Animal studies have indicated that the long-term administration of green tea may prevent age-related learning and memory decline by modulating the transcription factor cAMP-response element binding protein (CREB) and by upregulating synaptic plasticity-related proteins in the hippocampus [68, 69]. Similar memory-ameliorating effects were also shown in the context of neurodegenerative diseases, such as PD, AD, and multiple sclerosis [64].

Chlorogenic acid is a derivative of caffeic acid and is another common phenolic acid that is found in honey. A dose-dependent protective effect of chlorogenic acid against apoptosis was observed in pheochromocytoma-12 (PC12) cell lines that were exposed to methyl mercury-induced apoptotic damage. The protective activity of chlorogenic acid was associated with a reduction in the generation of reactive oxygen species (ROS) and the attenuation of apoptosis by the activation of caspase-3 [70]. In a study by Kwon et al. [71], the neuroprotective effects of chlorogenic acid on scopolamine-induced learning and memory impairment were investigated using several behavioral tests, such as the Y-maze, passive avoidance, and Morris water maze tests. Chlorogenic acid was found to significantly improve memory-related performance in all of the tests. It was concluded that chlorogenic acid may exert antiamnesic activity via the inhibition of acetylcholinesterase and malondialdehyde in the hippocampus and frontal cortex because chlorogenic acid inhibited the acetylcholinesterase activity of the hippocampus and frontal cortex in both* ex vivo* and* in vitro* model systems [71]. Chlorogenic acid inhibits the synthesis and release of inflammatory mediators, such as tumor necrosis alpha and nitric oxide (NO), thus contributing to anti-inflammatory and analgesic activities against carrageenan-induced inflammation [72]. Therefore, the chlorogenic acid in honey might have the capacity to attenuate neuroinflammation.

Chrysin (5,7-dihydroxyflavone) is another important flavonoid antioxidant that is present in honey. A behavioral experimental model revealed that chrysin is an anxiolytic that acts as a central receptor for benzodiazepine in instances where anxiety was reported to hamper cognitive function and learning capacity [73]. A study conducted by He et al. [74] showed that the therapeutic potential of chrysin in neurodegeneration-associated dementia resulted from cerebral hypoperfusion. The effects of chrysin were further investigated in a rat model of cognitive deficits and brain damage generated by the permanent occlusion of the bilateral common carotid arteries [74]. Such surgically induced hypoperfusion leads to a significant increase in the escape latency in the Morris water maze, with biochemical features of neural damage, such as increases in glial fibrillary acidic protein expression and apoptosis. Interestingly, chronic treatment with chrysin significantly alleviated neuronal damage and spatial memory deficits, with a reduction in lipid peroxidation and glutathione peroxidase activity but a decrease in SOD activity [74], indicating the neuroprotective role of honey.

p-Coumaric acid is the most abundant of the three hydroxy derivatives of cinnamic acid. A previous study demonstrated the oxidative stress reduction capacity and antigenotoxic capacity of p-coumaric acid [75]. In doxorubicin-induced cardiotoxicity, p-coumaric acid was able to increase the levels of GSH, SOD, and catalase activities with a concomitant reduction of lipid peroxidation [76]. p-Coumaric acid exhibited neuroprotective effects against 5-S-cysteinyl-dopamine-induced neurotoxicity. The extent to which p-coumaric acid confers neuroprotection was reported to be equal to or greater than that observed for the flavonoids (+)-catechin, (−)-epicatechin, and quercetin [77].

Ellagic acid is a phenolic acid that is found not only in fruits and vegetables but also in honey. In addition to its antioxidant activity, ellagic acid exerts chemopreventive effects, as indicated by its antiproliferative activity [78]. Interestingly, the chemopreventive effects of ellagic acid are executed through the reduction of oxidative stress at the cellular level [30]; moreover, oxidative stress is involved in neurodegeneration and age-related memory deficits. Hence, the probable neuroprotective effect of ellagic acid is promising. Treatment with ellagic acid also restores the levels of lipid peroxides and NO (nitric oxide), the activities of catalase and paraoxonase, and the total antioxidant status of the brain to normal levels [79]. Other experiments also support the hypothesis that ellagic acid reduces oxidative stress in the brain, which is reflected by improvements in cognitive function. Ferulic acid, another polyphenol that is found in honey, is a phenolic acid. Ferulic acid can provide neuroprotection against cerebral ischemia/reperfusion injury-associated apoptosis in rats. Ferulic acid treatment resulted in a decrease of the extent of apoptosis, with decreased levels of ICAM-1 mRNA and reduced numbers of microglia and macrophages. This phenomenon ultimately results in the downregulation of inflammation-induced oxidative stress and oxidative stress-related apoptosis [80]. In another study [81], the ameliorating effects of ferulic acid on apoptosis caused by cerebral ischemia or reperfusion were investigated. Ferulic acid was found to show neuroprotective effects against p38 mitogen-activated protein (MAP) kinase-mediated NO-induced apoptosis. It was also reported that ferulic acid inhibits Bax translocation, the release of cytochrome c, and p38 MAP kinase phosphorylation and enhances the expression of the GABAB1 receptor [81]. Ferulic acid could also alleviate learning and memory deficits through the concomitant inhibition of acetylcholinesterase activity and the augmentation of SOD activity while lowering the concentration of glutamic acid and malondialdehyde in the hippocampus of rats. These results suggested that the antioxidant activities of the honey may contribute to the improvement of the cholinergic system in the brain or to the inhibition of nerve injury by excitatory amino acids [82]. Ferulic acid may be useful for preventing trimethyltin-induced cognitive dysfunction as well as for boosting the activation of choline acetyltransferase (ChAT) in dementia [83].

*Gallic Acid.* Gallic acid prevents the apoptotic death of cortical neurons* in vitro* by inhibiting amyloid beta (25–35)-induced glutamate release and the generation of ROS [84]. Gallic acid possesses an antianxiolytic activity, which provided the primary evidence in support of the memory-ameliorating effect of gallic acid because anxiety is associated with memory disturbance [85]. The memory-ameliorating effects of gallic acid were further confirmed by Al Mansouri et al. [86], who revealed its neuroprotective effect on 6-hydroxydopamine-induced and cerebral oxidative stress-induced memory deficits. Gallic acid improved memory concomitant with increases in the total thiol pool and glutathione peroxide activity and decreased lipid peroxidation in the hippocampus and striatum [87]. However, we cannot claim that these biochemical findings are entirely responsible for the improvements in memory.

Kaempferol is a plant flavonoid that is also frequently found in honey. The toxicity of 1-methyl-4-phenyl-1,2,3,6-tetrahydropyridine (MPTP), which is a neurotoxin, leads to behavioral deficits, a depletion of dopamine, reductions in SOD and glutathione peroxidase activities, and an elevation of lipid peroxidation in the substantia nigra. The administration of kaempferol has been reported to reverse all of these behavioral and biochemical alterations and to prevent the loss of TH-positive neurons that is induced by MPTP (1-methyl-4-phenyl-1,2,3,6-tetrahydropyridine) [88]. In another study, kaempferol demonstrated the ability to protect primary neurons from rotenone-induced apoptotic challenge. Specifically, kaempferol-ameliorated antioxidant defenses and antiapoptotic effects involve the enhancement of mitochondrial turnover, which is mediated by autophagy [89]. Furthermore, kaempferol may be an optimal treatment for improving cognitive function due to its positive effects on depression, mood, and cognitive functions [90].

Luteolin is a flavonoid from the flavone class that has been reported to be found in honey. As is the case for most flavonoids, luteolin has antioxidant, anti-inflammatory, and antitumor properties [91]. Luteolin also has neuroprotective effects against microglia-induced neuronal cell death. The consumption of luteolin has been found to improve the spatial working memory of aged rats by mitigating microglia-associated inflammation in the hippocampus [92]. The impairment of learning acquisition induced by cholinergic neurotoxins and muscarinic and nicotinic receptor antagonists were reported to be attenuated by luteolin. This phenomenon, however, was not observed for dopaminergic neurotoxin- and serotonergic neurotoxin-induced memory impairments, thus confirming the involvement of the central cholinergic system in the memory-restoring function of luteolin [93].

Interestingly, Tsai et al. showed that the augmenting effect of luteolin on Mn-SOD and (Cu/Zn)-SOD activity as well as on the GSH levels in the cortex and hippocampus was associated with the amelioration of amyloid beta (1–40)-induced oxidative stress and cognitive deficits [94]. Luteolin is believed to enhance basal synaptic transmission and facilitate the induction of long-term potentiation (LTP) by high-frequency stimulation in the dental gyrus of the hippocampus. At the molecular level, the LTP inductive effect of luteolin involves the activation of cAMP response element-binding protein (CREB) [95].

Myricetin is another well-known flavonoid that has also been reported to be found in honey. Yasuo et al. (1994) demonstrated that myricetin can reduce the calcium-induced increase in oxidative metabolism in rat brain neurons when administered at a concentration of 3 nM or greater [96]. In the case of the retinoid-induced apoptosis of human neuroblastoma cells, myricetin induced neuroprotection through a protective effect against retinoid-induced oxidative stress. The neuroprotective effect of myricetin was reported to be associated with a reduction in lipid peroxidation, retinoid-induced hydrogen peroxide generation, and superoxide radical generation (O^2−^), as well as an elevation of the glutathione redox status [97]. In another study, myricetin was also reported to significantly prevent D-galactose-induced cognitive impairment. The results of this study also indicated that cognitive impairment was most likely mediated by the extracellular signal-regulated kinase- (ERK-) cyclic AMP response element binding protein (CREB) signaling pathway in the hippocampus [98].

Naringenin can confer a neuroprotective effect against quinolinic acid-induced excitotoxicity mediated by elevated intracellular calcium levels, NO-mediated oxidative stress, and, consequently, cell death [99]. Amyloid beta-protein-induced free radical-mediated neurotoxicity is also attenuated by naringenin [100]. Interestingly, free radical-mediated oxidative stress is a common manifestation of both amyloid beta- and quinolinic acid-induced neurotoxicity, and it has repeatedly been implicated in neurodegeneration and cognitive deficits. In a rat model, the administration of naringenin reversed the learning, memory, and cognitive impairments caused by the intracerebroventricular administration of streptozotocin [101]. Treatment with naringenin also increases the pool of GSH and the activities of glutathione peroxidase, glutathione reductase, glutathione-S-transferase, SOD, and choline acetyltransferase in the hippocampus in a rat model of Alzheimer's disease- (AD-) type neurodegeneration with cognitive impairment (AD-TNDCI), with a concomitant decrease in the loss of ChAT-positive neurons and impairments in spatial learning and memory [102].

Quercetin is another flavonoid with antioxidant activity that is commonly found in honey. An* in vitro* study demonstrates that quercetin can inhibit oxidative insults as well as oxidative stress-dependent and independent apoptosis in a neural cell model [103, 104]. Quercetin improves memory and hippocampal synaptic plasticity in models of memory impairment that is caused by chronic lead exposure [105]. Quercetin also exhibited neuroprotective effects against colchicine-induced cognitive impairments [106]. Another neuroprotective role confirmed for quercetin is the alleviation of neuroinflammation. According to Sharma et al., quercetin modulates an interleukin-1 beta-mediated inflammatory response in human astrocytes [107]. Quercetin also decreases the extent of ischemic injury in a lesion-repeated cerebral ischemic rat model and restores spatial memory through the suppression of hippocampal neuronal death [108, 109]. Interestingly, quercetin also showed ameliorating effects on the peripheral nervous system and the central nervous system (CNS). In another study, quercetin promoted the functional recovery of the spinal cord following acute injury [110].

## 6. Honey as a Neuroprotective Nutraceutical

Generally, neurodamaging insults are categorized as either endogenous or exogenous in nature. Because the neurons of the mature nervous system are postmitotic, they cannot be easily replaced by cell renewal; therefore, neuronal cell death is the most widely studied neuronal pathologies. Neurodegeneration describes the progressive loss of neural structure and function that culminates in neuronal cell death. Acute neurodegeneration is usually caused by a specific or traumatic event, such as cardiac arrest, trauma, or subarachnoid hemorrhage, whereas chronic neurodegeneration occurs within the context of a chronic disease state with a multifactorial origin, such as AD, PD, HD, or amyloid lateral sclerosis [111]. The biochemical events underlying neurodegeneration include oxidative stress, mitochondrial dysfunction, excitotoxicity, neuroinflammation, misfolded protein aggregation, and a loss of functionality [112]. The ultimate fate of such a neurodamaging insult is neuronal cell death through apoptosis, necrosis, or autophagy [113]. Therefore, oxidative stress, mitochondrial dysfunction, and inflammation are prime candidates for neuroprotection [114].

**Figure 1:**
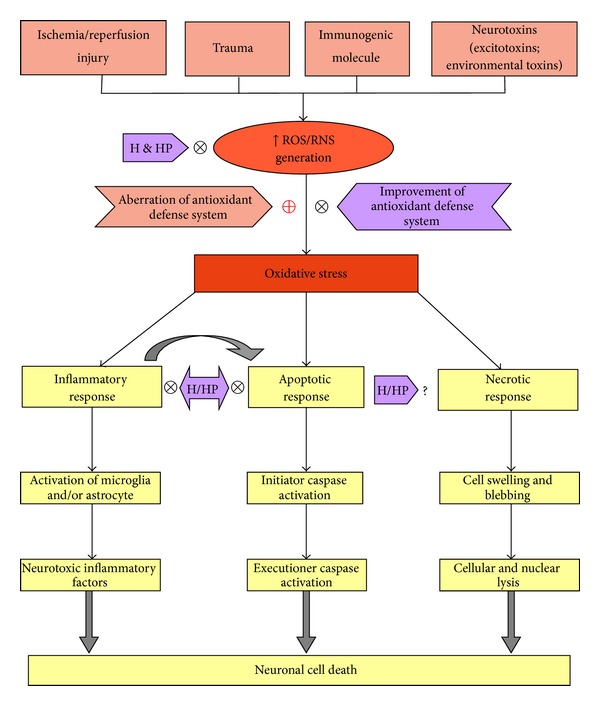
The putative neuroprotective mechanism of honey and its polyphenols. The generation of reactive oxygen species (ROS) and/or reactive nitrogen species (RNS) increases irrespective of neurodamaging insults that lead to oxidative stress. The dysfunction of the antioxidant defense system synergistically causes reactive species accumulation, leading to oxidative stress. The ultimate outcome of such oxidative stress is neuronal cell death through an inflammatory, apoptotic, or necrotic response [111–114, 116, 119]. Honey (H) and its polyphenol constituents (HP) can counter oxidative stress by limiting the generation of reactive species as well as by strengthening the cellular antioxidant defense system. Honey and several honey polyphenols (apigenin, ferulic acid, and catechin) prevent neuronal cell death by attenuating neuroinflammation and apoptosis. However, the neuroinflammatory responses overlap with apoptosis, and the role of honey in necrotic cell death remains unclear. X = stop or prevent and + = improve or intensify.

Much research over the last few decades has established nutraceuticals as neuroprotective agents. In addition to the acute modulation of the antioxidant defense system, several nutraceuticals can also modulate gene expression to confer long-term protection [115, 116]. Phytochemicals can also modify cellular behaviors by influencing receptor function as well as by modulating intracellular events, such as cell-signaling cascades [117, 118]. Honey and its constituents can ameliorate oxidative stress and oxidative stress-related effects. The neuroprotective effects of honey are exerted at different stages of neurodegeneration and play prominent roles in early events (Figure 1).

## 7. Honey as a Nootropic Nutraceutical

Learning and memory are the most exclusive and basic functions of the brain. Synaptic plasticity is thought to be crucial for information processing in the brain and underlies the processes of learning and memory [119]. Synaptic plasticity describes the capacity of neurons to change their efficiency in neuronal transmission in response to environmental stimuli and plays an essential role in memory formation. Long-term synaptic plasticity, or long-term potentiation (LTP), is the molecular analog of long-term memory and is the cellular model that underlies the processes of learning and memory [120, 121]. The induction, expression, and maintenance of LTP involve a series of biochemical events [122]. LTP is induced by the influx of calcium into postsynaptic neurons through a set of receptors and/or channels and is usually followed by the amplification of calcium levels due to the release of calcium from the Ca^2+^/InsP_3_-sensitive intracellular store [123, 124].

The expression of LTP involves the activation of several calcium-sensitive enzymes, which include calcium/calmodulin-regulated protein kinases (CaMKII and CaMKIV), the cAMP-dependent protein kinase A (PKA), protein kinase C (PKC), and MAPK/ERKs [125, 126]. Signaling events downstream and enzyme activation ultimately cause the initial expression and maintenance of LTP. However, the long-term expression and maintenance of LTP requires efficient gene expression. PKA may induce changes in the expression of genes via the phosphorylation of the transcription factor CREB. Phosphorylated CREB activates the transcription of genes with an upstream cAMP response element (CRE) [127]. The activation of CREB via MAPK/ERKs is thought to be connected to PKA and PKC signaling. Furthermore, CaMKII and CaMKIV may play a role in the maintenance of LTP through its effects on CREB phosphorylation [128]. Ultimately, CREB mediates the transcription and expression of at least two sets of genes, which include genes that regulate the transcription of other genes, such as* c-fos*,* c-jun*,* zif268*, and* Egr-3*, and effector genes, such as* Arc*,* Narp*,* Homer*,* Cox-2*, and* Rheb*, that directly act on cells to evoke different effects, including plastic changes [129].

**Figure 2:**
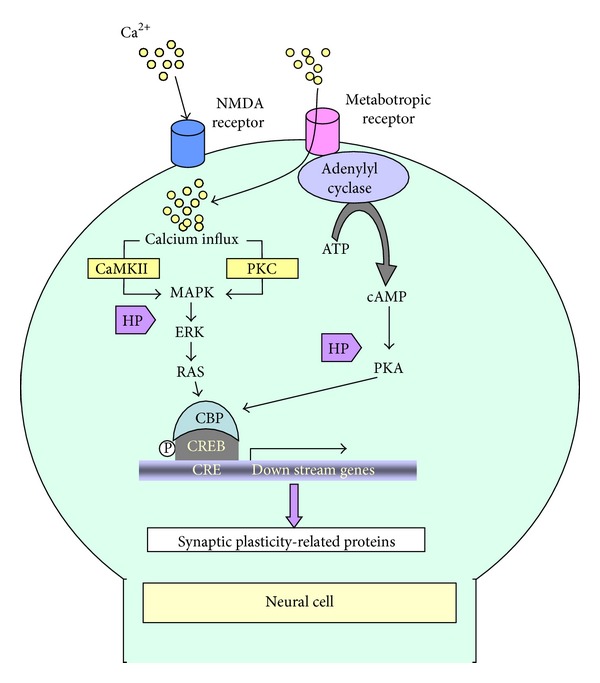
Putative nootropic mechanisms of honey and its polyphenols. Calcium influx via the N-methyl-D-aspartate receptor (NMDAR) occurs during the initial phase of NMDAR-dependent LTP. The inductive phase follows CREB phosphorylation through MAPK/ERKs signaling, which ultimately leads to the transcriptional regulation of synaptic plasticity-related proteins. Metabotropic receptors include ligand-gated ion channels that promote calcium influx (AMPA receptor) and enzyme-coupled receptors (such as cholinergic, glutamate, and dopamine receptors) that can trigger a second messenger (cAMP/cGMP) to activate downstream effector enzymes. The effector enzymes finally modulate the activation of CREB [123–128]. Honey polyphenols (HP: luteolin, myricetin, catechin) modulate synaptic plasticity through the activation of CREB by MAPK/ERKs and/or PKA-involved cellular signaling.

Current research has clarified only a portion of the involvement of honey polyphenols in memory-related signaling pathways. However, the overall body of knowledge clearly suggests the neuroprotective roles of honey and several supplementary experimental studies support its memory-improving effects. Overall, honey or its bioactive constituents might influence multiple signaling pathways to exert its memory-improving effects (Figure 2).

## 8. Concluding Remarks and Future Prospects

The brain is the supervisory organ with critical functions, such as body homeostasis maintenance, learning, and memory. Any neurodamaging insult leads to either the death or the functional aberration of neural cells, which results in neurodegeneration and the loss of motor function and the executive functions of the brain, such as memory. There is strong scientific support for the development of nutraceutical agents as novel neuroprotective therapies, and honey is one such promising nutraceutical antioxidant. However, past research paradigms did not evaluate the neuropharmacological and nootropic effects of honey using appropriately in-depth mechanistic approaches concerning biochemical and molecular interventions.

Honey has an appreciable nutritional value. Raw honey possesses anxiolytic, antinociceptive, anticonvulsant, and antidepressant effects and improves the oxidative status of the brain. Several honey supplementation studies suggest that honey polyphenols have neuroprotective and nootropic effects. Polyphenol constituents of honey quench biological reactive oxygen species that cause neurotoxicity and aging as well as the pathological deposition of misfolded proteins, such as amyloid beta. Polyphenol constituents of honey counter oxidative stress by excitotoxins, such as kainic acid and quinolinic acid, and neurotoxins, such as 5-S-cysteinyl-dopamine and 1-methyl-4-phenyl-1,2,3,6-tetrahydropyridine. Honey polyphenols also counter direct apoptotic challenges by amyloid beta, methyl mercury-induced, and retinoid. Raw honey and honey polyphenol attenuate the microglia-induced neuroinflammation that is induced by ischemia-reperfusion injury or immunogenic neurotoxins. Most importantly, honey polyphenols counter neuroinflammation in the hippocampus, a brain structure that is involved in spatial memory. Honey polyphenols also counter memory deficits and induce memory formation at the molecular level. Several studies suggest that the modulation of specific neural circuitry underlies the memory-ameliorating and neuropharmacological effects of honey polyphenols.

Our information demands the evaluation of the benefits of raw honey and its individual constituents in specific neurodegenerative diseases, such as AD, PD, and HD. The ultimate biochemical impact of honey on mitochondrial dysfunction, apoptosis, necrosis, excitotoxicity, and neuroinflammation should also be explored. Furthermore, exploration of the actual cell signaling cascades that are associated with synaptic plasticity may provide more specific therapeutic interventions using honey. The effect of honey on synaptic plasticity under normal and disease conditions should also be determined. The neural circuits and receptors that are involved in the neuropharmacological effects of honey, such as anxiolytic, antinociceptive, anticonvulsant, and antidepressant activities, should be examined in further detail.

## Figures and Tables

**Table 1:** An overview of composition of raw honey [22].

Nutrient	Value in 100 g
Moisture	17.10 g
Carbohydrate	82.40 g
Glucose	35.75 g
Fructose	40.94 g
Sucrose	0.89 g
Maltose	1.44 g
Galactose	3.10 g
Total dietary fiber	0.20 g
Protein	0.30 g
Total lipid (fat)	0.00 g
Ash	0.20 g
Energy	304 kcal

**Table 2:** A comparison of the minerals found in honey (major and essential trace minerals) with RDI as reported from several studies [25–27].

Major minerals	RDI	One tablespoon (21 g)	Essential trace minerals	RDI	One tablespoon (21 g)
Calcium	1000 mg	5.0 mg [27]	Copper	2 mg	0.4 mg [27]
Chloride	3400 mg	11.5 mg [25]	Fluoride	150 *μ*g	280.0 *μ*g [25]
Magnesium	350–400 mg	1.4 mg [26]	Iron	15–18 mg	4.6 mg [27]
Phosphorus	1000 mg	0.5 mg [26]	Molybdenum	75 *μ*g	4.0 *μ*g [25]
Potassium	3500 mg	21.0 mg [27]	Selenium	70 *μ*g	104.0 *μ*g [27]
Sodium	2400 mg	2.5 mg [26]	Zinc	15 mg	1.3 mg [27]

The values are daily reference values (DRVs) of RDI. The DRVs for major minerals are based on a caloric intake of 2,000 calories for adults and children (of four or more years of age). For trace elements, the RDIs that are given are the maximums for all sex and age groups [130, 131].
